# Protective effect of Pterostilbene against free radical mediated oxidative damage

**DOI:** 10.1186/1472-6882-13-238

**Published:** 2013-09-26

**Authors:** Jhankar D Acharya, Saroj S Ghaskadbi

**Affiliations:** 1 Department of Zoology, University of Pune, 411 007 Pune, India

**Keywords:** Anti-oxidant activity, Free radicals, Oxidative stress, Pterostilbene

## Abstract

**Background:**

Pterostilbene, a methoxylated analog of Resveratrol, is gradually gaining more importance as a therapeutic drug owing to its higher lipophilicity, bioavailability and biological activity than Resveratrol. This study was undertaken to characterize its ability to scavenge free radicals such as superoxide, hydroxyl and hydrogen peroxide and to protect bio-molecules within a cell against oxidative insult.

**Methods:**

Anti-oxidant activity of Pterostilbene was evaluated extensively by employing several *in vitro* radical scavenging/inhibiting assays and pulse radiolysis study. In addition, its ability to protect rat liver mitochondria against tertiary-butyl hydroperoxide (TBHP) and hydroxyl radical generated oxidative damage was determined by measuring the damage markers such as protein carbonyls, protein sulphydryls, lipid hydroperoxides, lipid peroxides and 8-hydroxy-2'-deoxyguanosine. Pterostilbene was also evaluated for its ability to inhibit •OH radical induced single strand breaks in pBR322 DNA.

**Result:**

Pterostilbene exhibited strong anti-oxidant activity against various free radicals such as DPPH, ABTS, hydroxyl, superoxide and hydrogen peroxide in a concentration dependent manner. Pterostilbene conferred protection to proteins, lipids and DNA in isolated mitochondrial fractions against TBHP and hydroxyl radical induced oxidative damage. It also protected pBR322 DNA against oxidative assault.

**Conclusions:**

Thus, present study provides an evidence for the strong anti-oxidant property of Pterostilbene, methoxylated analog of Resveratrol, thereby potentiating its role as an anti-oxidant.

## Background

Several polyphenols are well-known in inducing an array of biological responses in animal cells such as modulation of intercellular signaling, altered gene expression, protection against oxidation and enzyme inhibition. Among different classes of polyphenols, Stilbenoids, low molecular weight phenolics, are present in a very small proportion, and have not been studied to a great extent until recently. Resveratrol was first such stilbene to be found and isolated, and has been studied extensively since then for various activities such as anti-cancer, anti-oxidant, anti-inflammatory and several other beneficial activity [1].

Pterostilbene (trans-3,5-dimethoxy-4'-hydroxystilbene) is another such stilbene which is a naturally occurring methoxylated analog of Resveratrol and owing to its close structural similarity with Resveratrol, has gained importance [2]. Replacement of two hydroxyl groups with methoxy groups increases its oral bio-availability, and biological activity and slows its metabolism thereby prolonging its activity. It has been reported to be identified from various plant sources like the heartwood of *Pterocarpus santalinus*[3], *Pterocarpus marsupium*[4], leaves of *Vitis Vinifera*[5] and in healthy and immature berries of Pinot Noir and Gamay [6]. *Pterocarpus marsupium* Roxb is commonly known as the Indian kino tree (known in the vernacular as “Vijaysar” of “Bijasar”) and commonly grows in the central, western, and southern parts of India and in Sri Lanka. Its bark is used as an astringent, anti-diarrheal, and antacid [7] and is also effective in β-cell regeneration [8]. *P. marsupium* wood water has been reported to have hypoglycaemic activity in diabetic patients [9] and extract of its heartwood was found to be effective in reducing plasma glucose levels in newly-diagnosed diabetic patients [10]. Darakchasava, an ayurvedic medicine, is a well-known Indian herbal preparation of *Vitis Vinifera* and has been cited to have Pterostilbene as a major phenolic compound in it at a concentration of 6.8 mg/l whereas Resveratrol was present at a concentration of 1.3 mg/l [11]. Pterostilbene is attributed to have anti-diabetic [12-14], anti-cancer, anti-inflammatory [15-18] and anti-obesity [19] activity and is known to have anti-oxidant activity [2,20] comparable to Resveratrol [21]. Although anti-oxidant activity of Pterostilbene has been reported in terms of its ability to scavenge DPPH, ABTS and peroxyl radical, there exist scanty evidences in support of this view, and detailed analysis of its anti-oxidant activity needs to be evaluated.

Therefore, the present study was undertaken to characterize the free radical scavenging/inhibiting activity of Pterostilbene against an array of free radicals generated *in vitro.* In addition to this, its ability to confer protection to bio-molecules (lipids, proteins and DNA) from oxidative damage was also checked.

## Methods

### Chemicals and biologicals

2,2′- azobis-3-ethylbenzthiazoline-6-sulfonic acid (ABTS) diammonium salt, 1,1′-diphenyl-2- picrylhydrazyl (DPPH), ferric chloride, myoglobin, potassium ferricyanide, and 2-thiobarbituric acid (TBA) were purchased from Sigma Chemical Co., USA. All other chemicals used were purchased from Sisco Research Laboratories, India, Hi-MEDIA, India or Merck, Germany. Pterostilbene was purchased from Sigma, USA.

Pulse radiolysis study was done using linear accelerator (LINAC) electron pulse radiolysis system at the National Center for Free Radical Research, Pune University Campus, Pune, India.

Three months old male or female Wistar rats weighing 250 ± 20 g were used to isolate rat liver mitochondria and were procured from animal house facility of Institute of Veterinary and Biological Sciences, Pune. They were housed in polypropylene cages maintained at 25 ± 2°C with 12:12 h light and dark cycle. They were given feed and water *ad libitum*. Prior approval was obtained from the Pune University Institutional Animal Ethical Committee for the protocols used involving animals (Registration no. 538/02/c/CPCSEA).

### Methods

 A. *In vitro* radical scavenging assays

 Different concentrations of Pterostilbene (0.05, 0.10, 0.15 and 0.20 mM) were used for different radical scavenging/inhibiting assays. L-ascorbic acid was used as a standard and the activity of Pterostilbene was expressed as ascorbic acid equivalent anti-oxidant capacity (AEAC). DPPH radical scavenging activity was carried out according to the method of Aquino et al. [22]. In this method, DPPH•, a stable free radical having an absorption maximum at 515 nm was used which disappears on reduction in the presence of an anti-oxidant. The ferric reducing ability of Pterostilbene was measured using ferric reducing ability of plasma assay (FRAP) assay [23]. In this method ferric to ferrous ion reduction at low pH led to the formation of ferrous-tripyridyltriazine complex which was measured at 593 nm. Total anti-oxidant capacity (TAC) was estimated using the ferrylmyoglobin/ABTS method [24] which quantitates the relative ability of anti-oxidants to scavenge the 2,2′-azinobis(3-ethylbenzothiazoline-6-sulfonate) radical cation (ABTS^.+^). Hydroxyl radical scavenging activity was assayed according to the method of Halliwell et al. [25] by deoxyribose degradation method. H_2_O_2_ scavenging assay was performed according to the method of Nabavi et al. [26]. In this assay decrease in H_2_O_2_ concentration in the presence of different concentrations of Pterostilbene was measured at 240 nm. Superoxide radical scavenging activity was assayed spectrophtometrically by xanthine/xanthine oxidase according to Ukeda et al. [27] where the extent to which Pterostilbene inhibits the reduction of nitroblue-tetrazolium (NBT) by superoxide radical was monitored at 560 nm and expressed as % radical inhibition.

A.1. Pulse radiolysis

The ability of the Pterostilbene to scavenge ABTS^.+^, CO_3_•^–^ and •OH radicals was determined by pulse radiolysis. ABTS^.+^ radical was produced by reacting radiolytically generated azide radicals with ABTS. CO_3_•^–^ radicals were generated using a reaction mixture containing NaHCO_3_ and Na_2_CO_3_ saturated with nitrous oxide (N_2_O). In the presence of Pterostilbene, the decay of ABTS^.+^ and CO_3_•^–^ were monitored and correlated with the concentration of ascorbic acid equivalents [28].

Scavenging of •OH was determined by competition kinetic methods. Irradiation of water with 7 MeV electron pulse (50 ns pulse width) and dose rate of 17 Gy/pulse generates hydroxyl radicals, hydrated electrons and hydrogen atoms. To measure only the reactions of the •OH, all solutions were pre-saturated with N_2_O to remove dissolved oxygen gas and to quantitatively convert the hydrated electrons and hydrogen atoms to •OH. The ability of Pterostilbene to scavenge hydroxyl radicals was analysed by competition kinetics using potassium thiocyanate (KSCN) as a standard. In this method, •OH is made to react with KSCN in the absence and/or in the presence of different concentrations of Pterostilbene. •OH reacts competitively with SCN to produce (SCN)_2_•– which absorbs at 480 nm. In the presence of different concentrations of Pterostilbene, decrease in the formation of (SCN)_2_•– was measured. The difference between rate constant of (SCN)_2_•– in presence and absence of Pterostilbene was calculated [29,30]. The rate of hydroxyl radical scavenging was the slope of linear plot of this difference versus concentration of Pterostilbene.

 B. Isolation of rat liver mitochondria and exposure to oxidative stress

 Three-months old female Wistar rats were used for the preparation of mitochondria as described previously [31]. Briefly, rat liver was excised, and homogenized in 0.25 M sucrose containing 1 mM EDTA. The homogenate was centrifuged at 3000 *g* for 10 mins to remove cell debris and the nuclear fraction. The resultant supernatant was centrifuged at 10,000 *g* for 10 mins to sediment mitochondria. This pellet was washed with 5 mM phosphate buffer, pH 7.4. Protein was estimated by the method of Lowry et al. [32] and pellet was suspended in the sucrose-EDTA buffer.

 Isolated mitochondria were exposed to oxidative damage induced by tertiary butyl hydroperoxide (TBHP) and ascorbic acid and iron containing system (As-Fe^2+^) [33]. Briefly, mitochondria corresponding to 500 μg of protein were incubated with 400 μM TBHP and As-Fe^2+^ in the presence or absence of different concentrations of Pterostilbene for 60 mins at 37°C. The treated mitochondria were checked for oxidative damage caused to lipids, proteins and DNA.

B.3. Oxidative damage to DNA

Estimation of 8-hydroxy-2'-deoxyguanosine (8-OHdG)

Mitochondrial DNA was isolated using Nucleospin Tissue kit (Macherey-Nagel, USA) and quantitated on a nanophotometer. 8-OHdG concentration in DNA was determined by competitive ELISA using monoclonal antibody against 8-OHdG following the protocol of Modak et al. [37] and expressed as ng of 8-OHdG per 100 ng DNA.

B.3. Oxidative damage to lipids

**TBARs** were quantitated following the protocol of Devasagayam et al. [31] with slight modifications. Mitochondrial fractions after induction of oxidative damage were made to react with TBA reagent, boiled for 1 hour and the pink color developed due to TBARs formed was quantitated at 532 nm spectrophotometrically and expressed as nmoles of malondialdehyde (MDA) equivalents formed/ml after accounting for appropriate blanks.

**Lipid hydroperoxides** were estimated using FOXII reagent following the protocol of Nourooz-Zadeh et al. [36]. The working FOXII reagent was calibrated against known concentrations of hydrogen peroxide. Samples were made to react with FOXII reagent and incubated for 45 mins at 37°C, spun and absorbance was read at 560 nm.

B.3. Oxidative damage to Proteins

**Protein carbonyls** were quantitated based on the reaction of carbonyl groups with 2,4-dinitrophenylhydrazine (DNPH) to form a 2,4-dinitrophenylhydrazone, which can be measured at 365 nm [34]. The difference in the absorbance between blank and corresponding experiment gives the amount of carbonyl formed and was expressed as nmoles of protein carbonyls formed per milligram protein.

**Protein sulphydryls** were quantitated using Ellman’s reagent (5,5-dithiobis-2-nitrobenzoic acid) and expressed as nmoles protein sulphydryls per mg protein [35].

 C. Inhibition of single strand breaks in pBR322 plasmid DNA against oxidative damage

 pBR322 DNA was exposed to •OH radicals generated by the addition of hydrogen peroxide and ferrous sulphate in the presence and/or absence of different concentrations of Pterostilbene. Single strands generated in plasmid DNA were measured by quantitating the conversion of supercoiled DNA to nicked circular form, according to the procedure described by Zhao et al. [38]. Following incubation, the samples were immediately loaded onto 1% agarose gels and electrophoresed. The gel was stained with ethidium bromide and the fluorescence was observed under UV and the intensity of the bands was measured using Gel Documentation System (Alpha Innotech Corporation, USA).

### Statistical analysis

All the experiments were repeated four times and the data represents average of these sets with standard error. One–way analysis of variance (ANOVA) followed by post-hoc test was performed to determine the statistical significance of the differences among different experimental groups using SPSS 17.0 software. P value less than 0.05 was considered statistically significant and was indicated by dissimilar alphabets a, b, c, d and e in superscript.

## Results

### High anti-oxidant activity of Pterostilbene

**Figure 1:**
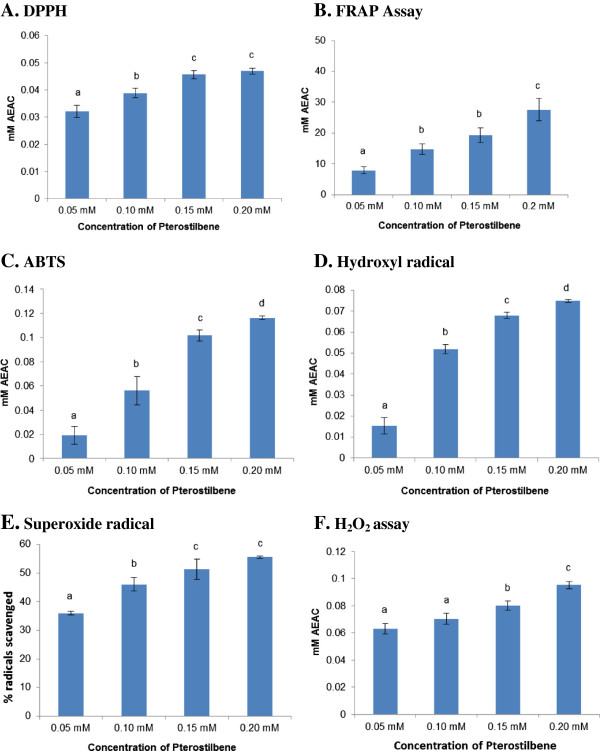
**Radical scavenging/inhibition by Pterostilbene. (A)** DPPH, **(B)** FRAP Assay, **(C)** ABTS, **(D)** Hydroxyl radical, **(E)** Superoxide radical and **(F)** H_2_O_2_ assay.

The anti-oxidant activity of Pterostilbene was estimated by performing several biochemical assays which determine either its free radical scavenging activity or its ability to inhibit formation of free radicals at different concentrations (0.05, 0.1, 0.15 and 0.2 mM). In DPPH radical scavenging assay, Pterostilbene exhibited concentration dependent activity from 0.05 mM to 0.15 mM Pterostilbene, with no further increase at 0.2 mM (Figure 1A). Pterostilbene also exhibited ability to reduce ferric to ferrous in a concentration dependent manner as assayed by FRAP assay (Figure 1B) with a maximum value of 27.5 ± 3.62 mM AEAC at 0.2 mM concentration. Figure 1C shows ability of Pterostilbene to inhibit ABTS radical formation which was assayed using ferrylmyoglobin/ABTS^.**+**^ detection method. Pterostilbene demonstrated concentration dependent inhibition of ABTS radical formation with a maximum value of 0.11 ± 0.001 mM AEAC at 0.2 mM concentration of Pterostilbene.

Superoxide radical scavenging activity of Pterostilbene was estimated by nitroblue tetrazolium reduction by xanthine-xanthine oxidase method. At the maximum concentration used Pterostilbene resulted in reduction of superoxide radicals by 55.6 ± 0.5% (Figure 1E). Similarly, Pterostilbene also exhibited concentration dependent increase in its ability to scavenge hydroxyl radical and hydrogen peroxide (Figure 1D and 1 F) with maximum ability of 0.09 ± 0.001 mM AEAC at a concentration of 0.2 mM.

**Figure 2:**
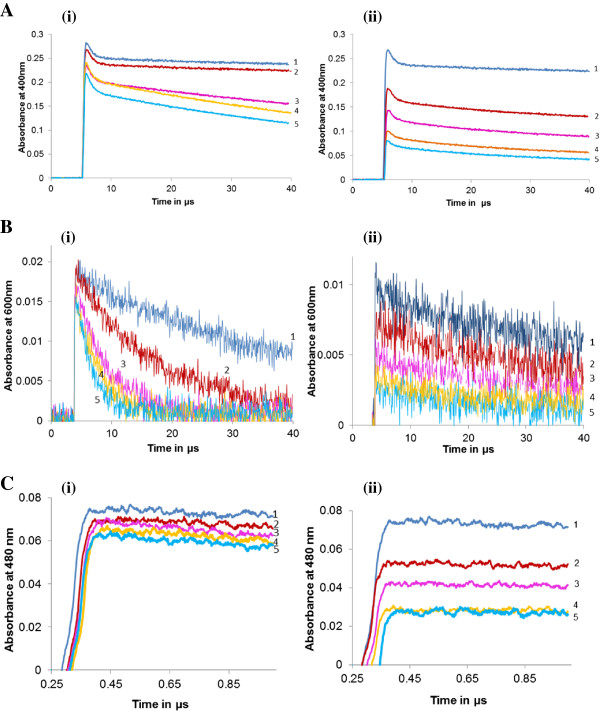
**Radical scavenging assays by pulse radiolysis.** Decay curve for ABTS^.+^ radical **(A)** and CO_3_•^–^ radical **(B)** in presence of different concentrations of (i) Ascorbic acid (1: Control, 2–5: 0.1, 0.2, 0.3 and 0.4 mM) and (ii) Pterostilbene. (1: Control, 2–5: 0.05, 0.1, 0.15 and 0.20 mM). Hydroxyl radical scavenging activity **(C)** by competitive kinetics in presence of different concentrations of (i) Ascorbic acid (1: Control, 2–5: 0.1, 0.2, 0.3 and 0.4 mM) and (ii) Pterostilbene. (1: Control, 2–5: 0.05, 0.1, 0.15 and 0.20 mM).

In addition to the above mentioned biochemical radical scavenging assays, Pulse radiolysis, a sensitive method to study scavenging of free radicals was employed. The linear plot of pseudo first order rate constant (K_abs_) versus ascorbic acid concentration was used to calibrate the standard curve for different concentrations of Pterostilbene. Figure 2 shows decay curves for ABTS^.+^ and CO_3_•^–^ radicals. Pterostilbene exhibited concentration dependent increase in the ability to scavenge ABTS^.+^ and CO_3_•^–^ radical with a maximum activity at 0.2 mM with a value of 1.38 mM AEAC and 6.85 M AEAC, respectively (Table 1).

**Table 1:** **ABTS**^**.+ **^**and CO**_**3**_**•**^**–**^**radical scavenging activity measured by pulse radiolysis for different concentrations of Pterostilbene**

**Pterostilbene**	**ABTS•**^**+**^	**CO**_**3**_**•**^**–**^
	**mM AEAC**	**M AEAC**
**0.05 mM**	1.26 ± 0.14	0.05 ± 0.01
**0.10 mM**	1.32 ± 0.18	1.00 ± 0.07
**0.15 mM**	1.38 ± 0.11	3.75 ± 0.47
**0.20 mM**	1.42 ± 0.30	6.85 ± 0.87

The scavenging activity of ABTS^.+^ and CO_3_•^–^ radicals were measured in terms of ascorbic acid equivalent antioxidant capacities (AEAC).

### Protection conferred against oxidatively damaged bio-molecules

Ability of Pterostilbene to inhibit oxidative damage in isolated mitochondria was assessed by exposing them to two different oxidants: TBHP and As-Fe^2+^ generated hydroxyl radical in presence and or absence of Pterostilbene. Lipid peroxides and hydroperoxides were used as markers of oxidative damage to lipids. Pterostilbene exhibited concentration dependent reduction in the formation of lipid peroxides and hydroperoxides in response to damage by TBHP and As-Fe^2+^. In presence of 0.2 mM Pterostilbene the nmoles of MDA formed significantly reduced by 92.1 ± 0.1% (1.11 ± 0.00 nmoles MDA/mg protein) and 94.1 ± 8.1% (0.88 ± 0.07 nmoles MDA/mg protein) against TBHP and As-Fe^2+^ induced damage, respectively. Concentration of lipid hydroperoxides increased significantly after exposure to TBHP and As-Fe^2+^ and decreased by 65.7 ± 4.2% (229.6 ± 20.1 nmoles hydroperoxides/mg protein) and 82.5 ± 4.6% (180.3 ± 17.9 nmoles hydroperoxides/mg protein), respectively in the presence of 0.2 mM Pterostilbene (Table 2).

**Table 2:** **Inhibition of lipid oxidation by PTS was measured in terms of nmoles MDA/mg protein and nmoles of hydroperoxides/mg protein in TBHP and As-Fe**^**2+ **^**mediated oxidatively damaged rat liver mitochondria**

	**Lipid peroxides**		**Lipid hydroperoxides**	
	**(nmoles MDA/mg protein)**		**(nmoles hydroperoxides/mg protein)**	
	**TBHP**	**As-Fe**^**2+**^	**TBHP**	**As-Fe**^**2+**^
Control	0.95 ± 0.01^a^	0.79 ± 0.07^a^	152.9 ± 15.1^a^	154.1 ± 5.1^a^
Damage	3.01 ± 0.08^b^	2.45 ± 0.07^b^	379.7 ± 11.3^b^	289.2 ± 11.2^b^
0.05 mM	1.94 ± 0.08^c^	1.60 ± 0.07^c^	362.9 ± 1.3^b^	243.0 ± 7.2^c^
0.10 mM	1.52 ± 0.02^d^	1.18 ± 0.04^d^	338.5 ± 2.4^c^	220.4 ± 12.7^d^
0.15 mM	1.34 ± 0.01^e^	0.90 ± 0.02^a^	321.4 ± 12.4^a^	208.8 ± 7.4^d^
0.20 mM	1.11 ± 0.00^e^	0.88 ± 0.07^a^	229.6 ± 20.1^a^	180.3 ± 17.9^e^

Values are expressed as mean ± SE. Dissimilar alphabets in superscript indicate significant difference at p< 0.05.

Protein carbonyl group (PCG) and protein sulphydryl group (PSG) were used as markers of oxidative damage to proteins. PCG increased significantly under oxidative stress conditions induced by TBHP and As-Fe^2+^, which was reduced by 99.4 ± 0.5% (3.55 ± 0.01 nmoles protein carbonyls/mg protein) and 94.0 ± 8.8% (3.92 ± 0.16 nmoles protein carbonyls/mg protein), respectively, in presence of 0.2 mM Pterostilbene. Protein sulphydryl groups reduced significantly under oxidative damage induced by TBHP and As-Fe^2+^, which was restored to 90.2 ± 4.8% (0.15 ± 0.00 nmoles protein sulphydryls/mg protein) and 87.9 ± 4.4% (1.15 ± 0.01 nmoles protein sulphydryls/mg protein), respectively in the presence of 0.2 mM Pterostilbene (Table 3).

**Table 3:** **Inhibition of protein oxidation by PTS was measured in terms of nmoles of protein carbonyls/mg protein and nmoles of protein sulphydryls/mg protein in TBHP and As-Fe**^**2+ **^**mediated oxidatively damaged rat liver mitochondria**

	**Protein carbonyl groups (nmoles protein carbonyls/mg protein)**		**Protein sulphydryl groups (nmoles protein sulphydryls/mg protein)**	
	**TBHP**	**As-Fe**^**2+**^	**TBHP**	**As-Fe**^**2+**^
**Control**	3.54 ± 0.02^a^	3.78 ± 0.21^a^	0.16 ± 0.00^a^	1.18 ± 0.04^a^
**Damage**	5.73 ± 0.14^b^	5.87 ± 0.02^b^	0.12 ± 0.01^b^	0.91 ± 0.01^b^
**0.05 mM**	4.40 ± 0.14^c^	5.35 ± 0.26^c^	0.13 ± 0.01^c^	1.02 ± 0.03^c^
**0.10 mM**	4.02 ± 0.23^d^	4.73 ± 0.02^d^	0.14 ± 0.01^c^	1.04 ± 0.01^c^
**0.15 mM**	3.57 ± 0.23^a^	4.45 ± 0.21^d^	0.14 ± 0.01^d^	1.07 ± 0.01^d^
**0.20 mM**	3.55 ± 0.01^a^	3.92 ± 0.16^a^	0.15 ± 0.00^a^	1.15 ± 0.01^a^

Values are expressed as mean ± SE. Dissimilar alphabets in superscript indicate significant difference at p< 0.05.

Oxidative damage to DNA results in oxidation of bases and one such modified base which is used as marker of oxidative stress is 8-hydroxy-2'-deoxyguanosine (8-OHdG). Protection conferred to mitochondrial DNA by Pterostilbene against oxidative damage was measured in terms of decrease in the formation of 8-OHdG (Table 4). Oxidative damage induced by TBHP and As-Fe^2+^ led to an increase in the concentration of 8-OHdG which reduced significantly (97.0 ± 1.1% (0.003 ± 4.5E-04 nmoles of 8-OHdG/100 ng DNA) and 96.4 ± 0.0% (0.003 ± 7.9E-04 nmoles of 8-OHdG/100 ng DNA), respectively) in the presence of 0.2 mM Pterostilbene (p < 0.05).

**Table 4:** **Inhibition of DNA oxidation by PTS was measured in terms of nmoles 8-OHdG/100 ng DNA in TBHP and As-Fe**^**2+**^**mediated oxidatively damaged rat liver mitochondria**

	**nmoles 8-OHdG/100 ng DNA**	
	**TBHP**	**As-Fe**^**2+**^
**Control**	0.003 ± 7.2E-05^a^	0.003 ± 7.6E-05^a^
**Damage**	0.014 ± 1.0E-04^b^	0.013 ± 4.2E-05^b^
**0.05 mM**	0.007 ± 1.0E-03^c^	0.006 ± 7.9E-05^c^
**0.10 mM**	0.006 ± 9.9E-04^d^	0.006 ± 2.5E-06^c^
**0.15 mM**	0.005 ± 7.7E-04^e^	0.004 ± 1.1E-04^d^
**0.20 mM**	0.003 ± 4.5E-04^a^	0.003 ± 7.9E-04^a^

Values are expressed as mean ± SE. Dissimilar alphabets in superscript indicate significant difference at p< 0.05.

### Strong ability to inhibit single strand breaks in pBR322

**Figure 3:**
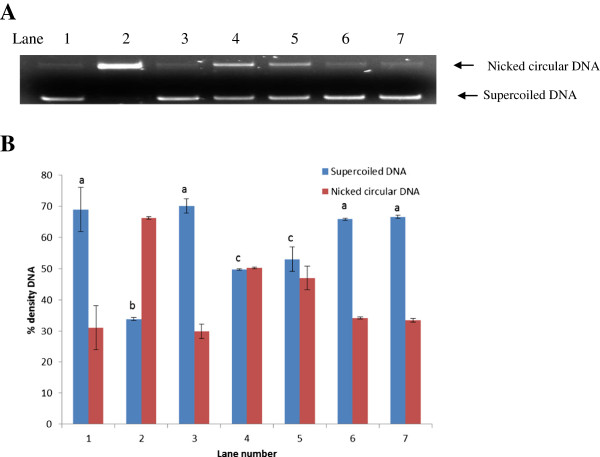
**A. Gel electrophoresis pattern of pBR322 plasmid DNA after induction of oxidative damage in the presence of different concentrations of Pterostilbene.** Lane 1-Undamaged DNA; Lane 2- Damaged DNA in presence of hydroxyl radical; Lane 3-DNA in presence of Pterostilbene alone; Lanes 4-7- oxidatively damaged DNA in presence of 0.05, 0.10, 0.15 and 0.20 mM Pterostilbene, respectively. **B.** Densitogram analysis of electrophoreogram to quantitate supercoiled and nicked circular DNA. Values are expressed as mean ± SE. Dissimilar alphabets in superscript indicate significant difference at p< 0.05.

Exposure to oxidative stress induces both single and double strand breaks converting the supercoiled pBR322 DNA into open circular form and linear form, respectively. Oxidative stress mediated DNA strand breaks were induced by generating •OH radicals through Fenton reaction. Plasmid DNA was exposed to ^.^OH radicals and was significantly damaged as seen in lane 2 (Figure 3A) where 66.2 ± 0.4% DNA was converted to nicked circular form compared to the untreated pBR322 in lane 1 where 68.9 ± 7.1% DNA remained in supercoiled form. Densitogram analysis (Figure 3B) revealed that in the presence of Pterostilbene alone, 70.1 ± 2.3% DNA remained in supercoiled form (lane 3) (p < 0.05). Pterostilbene inhibited the formation of single strand breaks after exposure to •OH radicals in a concentration dependent manner (Lanes 4–7).

## Discussion

Extract of *Pterocarpus marsupium*, an Indian medicinal plant, has been demonstrated to have anti-oxidant activity and also protected liver cells against ethanol induced oxidative stress [39]. Since Pterostilbene is one of the major active constituents in *Pterocarpus marsupium*, we speculated that Pterostilbene could have contributed to its anti-oxidant activity. Therefore, commercially available and purified Pterostilbene was studied with respect to its anti-oxidant activity. Earlier studies have reported anti-oxidant activity in terms of ability to scavenge DPPH and ABTS radicals; however, in this study we have evaluated comprehensively its anti-oxidant activity against a variety of free radicals. In addition to this, we have also assessed its ability to protect cellular bio-molecules against oxidative damage. The results obtained indicate that Pterostilbene possesses strong anti-oxidant activity. Ability to scavenge and/or inhibit formation of free radicals was assessed by performing different *in vitro* radical scavenging assays. The DPPH• radical scavenging assay corresponds to the primary radical scavenging activity of an anti-oxidant, whereas ferrylmyoglobin/ABTS^•+^ corresponds to the ability of an anti-oxidant to inhibit radical formation. Pterostilbene demonstrated ability to scavenge DPPH and inhibit ABTS radical formation in a concentration dependent manner which could be attributed to the ability of Pterostilbene to act as a donor of hydrogen atoms or electrons. Both, DPPH and ABTS radical scavenging activity of Pterostilbene is in accordance with the previous study [2,20]. The FRAP assay evaluates the reducing capacity of an anti-oxidant and Pterostilbene exhibited concentration dependent anti-oxidant potential. In biological systems, superoxide radicals are generated as a by-product of cellular respiration. Superoxide can generate hydroxyl radical by Haber Wiess reaction and produce H_2_O_2_ on dismutation by superoxide dismutase. Hydrogen peroxide is another biological reactive oxygen species, which at low concentration is necessary for cellular signaling, but at a high concentration it could be detrimental [40]. Under physiological conditions H_2_O_2_ is further decomposed into water and oxygen by catalase. However, during stress conditions excess production of free radicals overwhelms the anti-oxidant defense system. This necessitates supplementation with anti-oxidants to maintain redox homeostasis. In the present study, Pterostilbene exhibited ability to scavenge superoxide, hydroxyl and H_2_O_2_ in a concentration dependent manner with maximum activity at 0.02 mM(50 μg/ml). Recent reports suggest that in humans Pterostilbene upto a dose of 250 mg/day does not have any adverse toxic effects on hepatic and renal functions, thereby emphasizing its importance as a safe drug [41].

Free radicals generated *in vivo* as a result of cellular respiration have a very short half-life and the clearance rate of these radicals is very rapid. Taking this into consideration, pulse radiolysis was carried out to study reactions occurring on a time scale faster than one hundred microseconds and to study the kinetics of radical scavenging reactions. Pterostilbene exhibited ABTS^.+^, CO_3_•^–^ and •OH radical scavenging activity in pulse radiolysis study indicating its ability to react with radicals within a short span of time, as needed in biological systems.

Further, since mitochondria is a major sub-cellular organelle where the cellular system of energy provision is localised, rat liver mitochondria was used as a model system to study protective effect of Pterostilbene against TBHP and •OH like radical (generated by Fenton reaction) induced oxidative damage. ROS exposure leads to a reduction in mitochondrial membrane potential, oxidation of functionally important thiol groups on mitochondrial enzymes and induces cell death [42,43]. ROS degrades polyunsaturated fatty acids, incorporated in all biological membranes, to peroxyl radicals (ROO^.^) which is further converted to malondialdehyde (MDA) through a series of chain reactions. Damage to proteins is also of particular importance *in vivo* as it affects the functionality of several receptors, enzymes, transport proteins and contributes to secondary damage of other bio-molecules, e.g. by inactivating anti-oxidant defense enzymes or repair enzymes. ROS, especially hydroxyl radical is known to react with all the components of DNA, damaging both purine and pyrimidine bases and also the deoxyribose backbone [44] generating a wide range of oxidative DNA lesions, for example, 8-OHdG. This damage to bio-molecules within a cell results due to excessive ROS generation which overwhelms the intracellular defense mechanism. Therefore, dietary anti-oxidants capable of scavenging free radicals can prove to be beneficial in ameliorating these deleterious effects of ROS. Treatment with Pterostilbene, which in our study exhibited *in vitro* radical scavenging/inhibiting activity, also protected lipids, proteins and DNA in rat liver mitochondria from oxidative damage.

Additionally, ability of Pterostilbene to inhibit single strand DNA breaks was estimated using pBR322 DNA. Single strand breaks were induced by generating hydroxyl radical (by Fenton reaction) which converts the supercoiled DNA to nicked circular DNA. This damage was prevented in presence of different concentrations of Pterostilbene. This could be attributed to the fact that Pterostilbene scavenges hydroxyl radicals, as seen in this study, thereby protecting DNA against free radical assault. Additionally, polyphenols are also known as metal chelators and chelation of transition metals such as Fe^2+^ can directly reduce the rate of Fenton reaction, thereby protecting against damage by hydroxyl radicals [44].

Thus, the data obtained clearly shows that Pterostilbene has radical scavenging/inhibiting activity and also protects lipids, proteins and DNA against oxidative assault. Presence of a free 4-OH group in Pterostilbene is suggested to be crucial for its anti-oxidant activity [21].

## Conclusion

The results obtained suggest that Pterostilbene, an analog of Resveratrol, possesses strong free radical scavenging/inhibiting activity and also has the ability to protect bio-molecules in a cell against oxidative stress, thus potentiating its therapeutic role as an anti-oxidant.

## Competing interest

The authors declare that they have no competing interests.

## Authors’ contributions

JDA carried out all the experimental work, analysed the data and wrote the first draft. SSG supervised the study design and revised the manuscript. All authors read and approved the final manuscript.

## Pre-publication history

The pre-publication history for this paper can be accessed here:

http://www.biomedcentral.com/1472-6882/13/238/prepub
